# Functional Characterisation of the Maturation of the Blood-Brain Barrier in Larval Zebrafish

**DOI:** 10.1371/journal.pone.0077548

**Published:** 2013-10-16

**Authors:** Angeleen Fleming, Heike Diekmann, Paul Goldsmith

**Affiliations:** DanioLabs Ltd., Cambridge Research Park, Cambridge, United Kingdom; Institut Curie, France

## Abstract

Zebrafish are becoming increasingly popular as an organism in which to model human disease and to study the effects of small molecules on complex physiological and pathological processes. Since larvae are no more than a few millimetres in length, and can live in volumes as small as 100 microliters, they are particularly amenable to high-throughput and high content compound screening in 96 well plate format. There is a growing literature providing evidence that many compounds show similar pharmacological effects in zebrafish as they do in mammals, and in particular humans. However, a major question regarding their utility for small molecule screening for neurological conditions is whether a molecule will reach its target site within the central nervous system. Studies have shown that Claudin-5 and ZO-1, tight-junction proteins which are essential for blood-brain barrier (BBB) integrity in mammals, can be detected in some cerebral vessels in zebrafish from 3 days post-fertilisation (d.p.f.) onwards and this timing coincides with the retention of dyes, immunoreactive tracers and fluorescent markers within some but not all cerebral vessels. Whilst these findings demonstrate that features of a BBB are first present at 3 d.p.f., it is not clear how quickly the zebrafish BBB matures or how closely the barrier resembles that of mammals. Here, we have combined anatomical analysis by transmission electron microscopy, functional investigation using fluorescent markers and compound uptake using liquid chromatography/tandem mass spectrometry to demonstrate that maturation of the zebrafish BBB occurs between 3 d.p.f. and 10 d.p.f. and that this barrier shares both structural and functional similarities with that of mammals.

## Introduction

In mammals, the blood-brain barrier (BBB) provides a complex obstacle to the penetration of drugs into the central nervous system (CNS). The first level of barrier is presented by the tight junctions between endothelial cells of the vasculature. These high resistance tight junctions, made up by proteins such as claudin-5 and ZO-1, render brain capillary endothelia tightly sealed, in contrast to “leaky” endothelial capillaries in the periphery. Thus, there is no paracellular movement of fluid and only minimal pinocytosis from capillaries into the CNS [1]. The next level of barrier function is provided by capillary pericytes, which wrap around the endothelial cells of the capillary walls. Lastly, the outermost layer comprises astrocyte end feet which surround the endothelium and pericytes. In addition to providing a physical barrier, these three cell types express a variety of enzymes, such as aminopeptidases, carboxypeptidases, endopeptidases and cholinesterases, which inactivate many drugs [2], and in some cases, may also activate pro-drugs. In spite of these physical and enzymatic barriers, certain molecules are able to freely diffuse across the BBB, but are prevented from accumulating in the brain as they are actively effluxed by specific transporters, the most notable of which is P-glycoprotein (Pgp) [1]. Conversely, inwardly directed transporter systems, such as GLUT1/Slc2a1 (glucose transport), Slc7a1 and Slc7a5 (amino acids), low-density lipoprotein receptors (LRPs) and ion pumps, permit the entry of a variety of molecules that would otherwise be unable to enter the brain [1].

Zebrafish are popular as a vertebrate model with which to perform compound screens [3,4] and increasingly used to model human neurological disease processes, such as epilepsy and neurodegeneration [5,6]. Therefore, it is important to understand whether and when the zebrafish BBB forms and compare its characteristics and function with that of mammals. Adult zebrafish express the tight junction proteins ZO-1 and claudin-5 in the endothelial vascular cells within the brain [7]. In addition, size dependent exclusion of immuno-reactive compounds occurs in the adult zebrafish brain. For example, analysis of the distribution of enzymatically active compounds injected into the heart demonstrated that HRP (44kDa) was retained in cerebral vessels, whilst sulfo-NHS-Biotin (0.443kDa) diffused into the brain, indicating a size dependent exclusion mechanism [7]. Tight junctions play a major role in size-dependent exclusion in mammals and loosening of the size exclusion limit is observed in claudin-5 knockout mice [8]. Therefore, the presence of claudin-5 and ZO-1 in the adult zebrafish brain could account for these observed effects. These findings demonstrate that adult zebrafish possess a BBB but it is not known when this barrier becomes functional during zebrafish development. 

Expression of claudin-5 and ZO-1 have been detected in cerebral microvessels of larval zebrafish from as early as 2 and 3 days post-fertilisation (d.p.f.) respectively [7,9]. Furthermore, several studies have used fluorescent dyes or transgenically-encoded fluorescently-tagged plasma protein to demonstrate size dependent exclusion occurs from around 3 d.p.f. [7,9–11]. However, size exclusion seems to occur only in certain cerebral vessels at this age, while others are still “leaky”. In mammals, the BBB gradually matures during development, with permeability to small molecules decreasing with age (i.e. substances excluded from the adult rat brain do permeate embryonic brain capillaries) [12]. Similarly, the developing mouse BBB shows decreasing compound permeability as it matures [13]. 

In this study, we set out to investigate how size-dependent exclusion matures during larval development, to determine the degree of similarity in exclusion properties to those of mammalian BBB and to study the cellular components surrounding the vascular endothelium in the brain. We show that zebrafish do indeed gradually develop a sophisticated BBB with active transport systems. This allows for the design of rational strategies for screening compounds for neurological indications and interpretation of results, as well as potentially providing a system to predict whether a novel compound might penetrate the human brain.

## Methods

### Ethics statement

This work was licenced by the Home Office under the Animals (Scientific Procedures) Act (Home Office licence number PPL80/2074 for work performed at DanioLabs Ltd and PPL 80/2322 for work performed at the University of Cambridge). Protocols were approved by the local Animal Welfare Ethical Review Committee (AWERC); namely, DanioLabs AWERC and University of Cambridge AWERC. Appropriate steps were taken to ameliorate suffering in all work. Experiments were designed with the minimum number of animals necessary to produce meaningful results.

### Husbandry and experimental procedures

Embryos were collected from matings of adults of wildtype (TL, AB and WIK strains) and Tg(fli1a:EGFP)^y1^ transgenic fish [14] kept under standard conditions [15]. Embryos were reared in embryo medium (5 mM NaCl, 0.17 mM KCl,0.33 mMCaCl_2_, 0.33 mM Mg_2_SO_4_, 10^-5^% Methylene Blue) and staged using standard criteria [16]. All experiments were carried out in accordance with the Animals (Scientific Procedures) Act, 1986. 

### Dye exclusion experiments

Stocks of 2% Evans blue (961 Da) and 10% sodium fluorescein (376 Da) in 0.9% saline were prepared and stored at 4 °C. Larvae were anaesthetised by immersion in 0.2 mg/ml 3-amino benzoic acid (MS222) prior to being immobilised and positioned laterally in 3% methyl cellulose in embryo medium such that the heart was accessible. Larvae and juveniles from 2 days post-fertilisation (d.p.f.) to 30 d.p.f. were injected with 0.5 to 2 nl (depending on age) of dye or saline control into the pericardial region, using a standard zebrafish microinjection apparatus [15]. Fish were transferred to fresh embryo medium to recover from anaesthesia, then viewed using a fluorescence dissecting microscope at 1 hour intervals to determine the time of uptake of the dye into the circulation and the time at which dye had permeated from the circulation into non-vascular body tissue. Representative images of dye distribution *in vivo* were taken on a Zeiss AxioZoom V16 microscope with Apotome. Dye and saline injected larvae were anaesthetised at 4 or 6 hours after injection and fixed in 4% paraformaldehyde in PBS. Ten larvae per age group and injection treatment were embedded in Sakura Tissue-Tek OCT (Bayer, Newbury, UK) and frozen parasagittal sections were cut at 10 µm thickness. 

### Quantification of dye fluorescence

Sections were visualised without mounting medium by fluorescence microscopy on an Olympus BX51 microscope. Three midline sagittal sections from each larva were identified and images were captured using a ColorView camera (Olympus) and AnalySis software (Soft Imaging System). The fluorescence intensity within the brain was quantified for each midline section of each larva in each treatment group using colour threshold and area measurements in AnalySis. Mean values, standard deviations and Student’s t-tests were calculated using Excel software (Microsoft Office). 

### Transmission Electron Microscopy

Larvae were anaesthetised at 3, 5, 8 and 10 d.p.f. by immersion in 0.2 mg/ml MS222, fixed in 4% glutaraldehyde in cacodylate buffer containing 0.006% hydrogen peroxide for 3 hours and washed in cacodylate buffer before post-fixing in osmium tetroxide. Samples were bulk stained with uranyl acetate, dehydrated in ethanol and embedded in Spurr’s resin. Thin parasagittal sections (5 nm) were prepared with a Leica Ultracut UCT, stained with uranyl acetate and lead citrate and viewed in a Philips CM100 electron microscope at 80 KV.

### *In silico* sequence analysis

Protein sequences of mammalian and zebrafish ABCB gene family members were identified from ENSEMBL (human GRCh37; mouse GRCm38; zebrafish Zv9). BLAST searches were performed to identify whether un-annotated paralogues could be identified from the zebrafish genome. Sequence alignments were performed using ClustalW.

### Zebrafish abcb1/4/5 expression

Larvae were anaesthetised at 3, 5, 8 and 10 d.p.f. by immersion in 0.2 mg/ml MS222, fixed in 4% PFA and processed for wholemount antibody staining as previously described [17]. Immunohistochemistry was performed with an antibody raised against mouse ABCB1 (Covance) at 1:100 dilution and the staining detected using Alexa568 fluorescent secondary antibody (Molecular Probes). Larvae were mounted in chamber slides in Prolong Gold antifade mounting medium (Invitrogen) and viewed using a Zeiss StereoZoom V16 microscope under fluorescence illumination. Optical sectioning was performed using an Apotome.2 (Zeiss) and representative images were captured using and AxioCam MR digital camera (Zeiss) and Zen 2012 software (Zeiss).

### Drug exposure and distribution experiments

Initial drug exposure assays were performed in larval zebrafish to identify a sufficiently high concentration of drug that could be detected by liquid chromatography**/**tandem mass spectrometry (LC/MS/MS) analysis without causing toxicity. Maximum tolerated concentration (MTC) was determined using n=6 larvae per group. A non-toxic drug concentration of 15 µg/ml was selected for all subsequent compound exposure experiments except for scopolamine, which was used at 5 µg/ml. 60 larvae (equivalent to approximately 20 mg wet tissue) were transferred into a single well of a 12 well plate (Corning) and the final volume of embryo medium per well was adjusted to 1.5 ml. Compounds of interest were prepared as 2 mg/ml stocks in dimethyl sulfoxide (DMSO). 11.25 µl of stock was added to each well, except for scopolamine (3.75 µl). Larvae were incubated in the drug for 1 hour at room temperature then anaesthetised on ice. For whole uptake analysis, anaesthetised larvae were transferred to 1.5 ml microfuge tubes and all excess liquid was removed. 100 µl ice cold PBS was added, samples were briefly centrifuged and all excess liquid was removed. For brain versus body uptake analysis, larvae were anaesthetised by chilling prior to decapitation using fine iridectomy scissors and No. 5 watchmaker forceps. Head and body tissue samples were collected in separate microfuge tubes on ice and excess liquid removed. 100 µl ice cold PBS was added and samples were briefly centrifuged and all excess liquid removed. The weight of the tissue sample was measured and the samples were frozen at -20 °C prior to extraction for LC/MS/MS. 

For studies investigating the effect of Abcb1/4/5 efflux on drug distribution, larvae were exposed to the ABCB1/4/5 efflux inhibitor verapamil at 50 µg/ml, a concentration previously demonstrated to be non-toxic, for 2 hours prior to exposure to the test compound. Test compound was added to the well containing 50 µg/ml verapamil and larvae were incubated for a further hour at room temperature prior to tissue collection as described. For subsequent LC/MS/MS analysis, tissue samples were thawed and after addition of an internal standard (clozapine) extracted into an organic solvent (tertiary-butyl methyl ether, t-bme). The solvent extract was then evaporated and reconstituted in methanol/acetic acid, except for scopolamine exposed samples, which were reconstituted in acetonitrile/ammonia. Liquid chromatography for haloperidol, desloratadine and diphenhydramine (100 µl injections of extracted samples) was carried out using a Phenomenex Luna C18(2), 50 x 2 mm, 5 µm analytical column with a mobile phase gradient given in Table S1. Chromatography for scopolamine and scopolamine N-butyl bromide was performed using a Restek Ultra IBD, 150 x 3.2 mm, 5 µm analytical column with a mobile phase gradient given in Table S2. The analytes and internal standard were ionised under atmospheric pressure chemical ionisation (APCI) conditions operating in positive ion mode. Detection was via tandem mass spectrometry (MS/MS) using selected ion monitoring (SIM). MS/MS scan parameters under APCI conditions are given in Table S3. All analyses were performed using a Finnigan LCQ quadrupole ion trap mass spectrometer. The ratio of the amount of drug found in the trunk relative to that in the head was calculated. Changes in trunk/head drug ratios of greater than 20% were considered to be indicative of altered distribution.

### The effect of Pgp inhibitors on the distribution of rhodamine 123

Rhodamine 123 (R123) was dissolved in 10% ethanol/0.9% saline to make a 0.5 mg/ml stock. Dye exclusion experiments on larvae at 3, 5, 8 and 10 d.p.f. were performed as described above using 0.2 nl R123. Some larvae were exposed to verapamil at 50 µg/ml for 2 hours prior to and during intrapericardial injection of R123 and for 3 hours after injection. At 3 hours after injection, larvae were anaesthetised and fixed in 4% PFA prior to processing for frozen sections. Quantification of fluorescence in the brain was performed as described above. 

### Analysis of GFAP expression

Tg(fli1a:EGFP)^y1^ larvae at 3, 5, 7 and 10 d.p.f. were fixed in 4 % PFA, then processed for antibody staining as previously described [17]. The GFAP antibody (zrf-1, ZIRC) was used at 1:25 dilution and detected with Alexa568 fluorescent secondary antibody. Larvae were mounted in chamber slides in Prolong Gold antifade mounting medium (Invitrogen) and viewed using a Zeiss StereoZoom V16 microscope under fluorescence illumination. Optical sectioning was performed using an Apotome.2 (Zeiss) and representative images were captured using and AxioCam MR digital camera (Zeiss) and Zen 2012 software (Zeiss).

## Results

### Developmental changes in the size of fluorescent molecules excluded from the CNS

**Figure 1:**
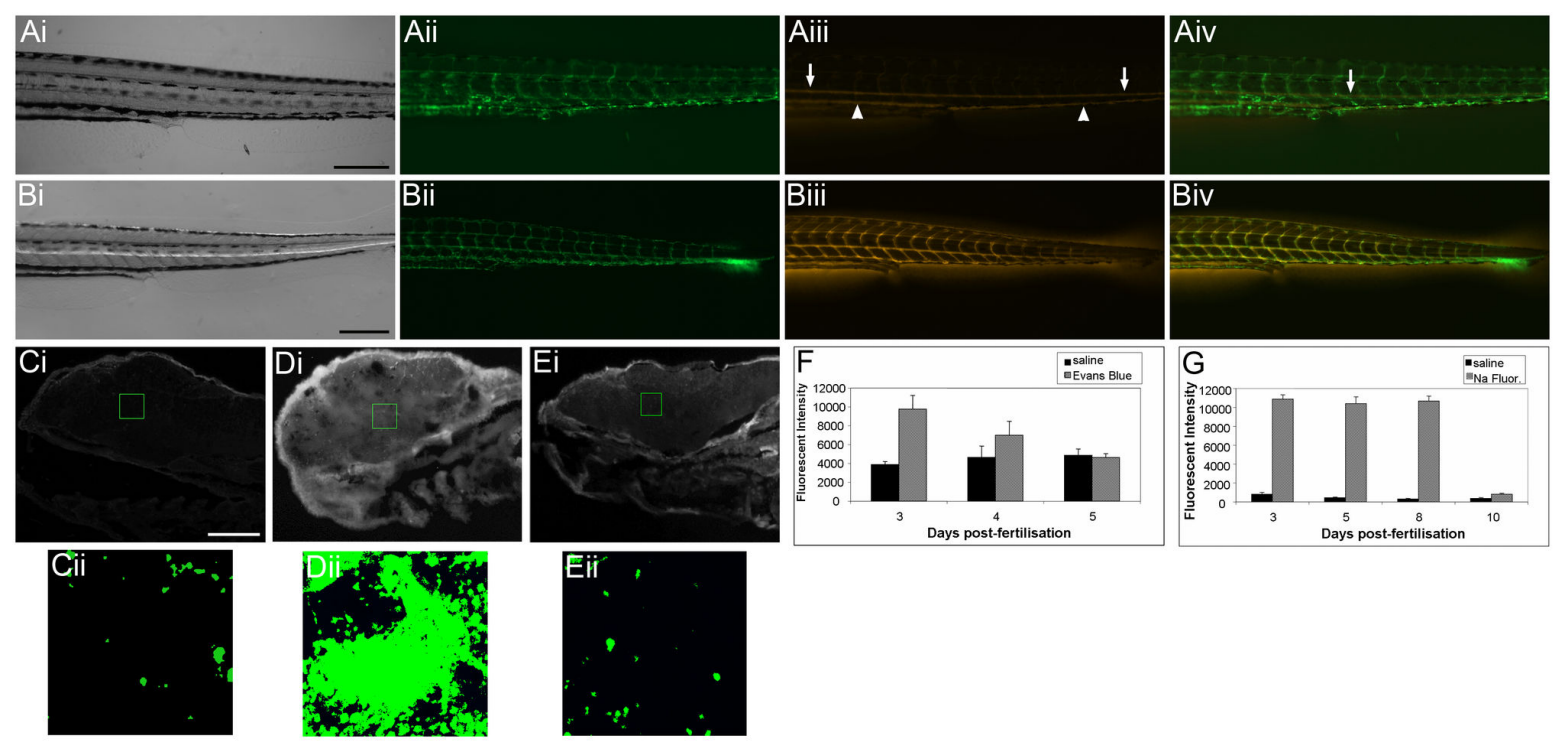
Investigation of blood-brain barrier maturation using fluorescent dyes. Ai-iv) One hour after pericardial injection of Evans blue into 8 d.p.f. Tg(fli1a:EGFP)^y1^ larvae, strong Evan’s blue fluorescence could be observed in the dorsal aorta (arrows) and vena cava (arrowheads) and weak fluorescence could be observed in individual segmental vessels of the trunk. Bi-iv) 4 hours after injection of Evan’s blue, strong fluorescence is still observed in dorsal aorta and vena cava and additionally in segmental vessels. In addition, fluorescence is also observed in trunk muscles between the segmental vessels and in the fin mesenchyme demonstrating that the dye has penetrated into surrounding tissue. Ci) Parasagittal section of a 3 d.p.f. zebrafish larva 3 hours after pericardial injection with saline control. Cii) High magnification fluorescent imaging of the region of marked in Ci). Di) Parasagittal section of a 3 d.p.f. zebrafish larva 3 hours after pericardial injection with Evan’s blue. Dii) High magnification fluorescent imaging of the region of marked in Di). Ei) Parasagittal section of a 5 d.p.f. zebrafish larva 3 hours after pericardial injection with Evan’s Blue. Cii) High magnification fluorescent imaging of the region of marked in Ci). Cii – Eii) The fluorescent intensity of dye within the brain was quantified using image thesholding (pseudo-coloured green) and area over threshold was measured using AnalySis software. F & G) The fluorescence intensity of injected dyes was measured in the brain of zebrafish following peripheral injection at various time points of zebrafish development. Graphs show mean fluorescent intensity (± std dev.) for each treatment. F) Evans blue, (961 Da) a large molecule known to form multimers with serum proteins, is excluded from the brain from day 5. G) Sodium fluorescein (376 Da) permeates into the brain until 8 d.p.f. but is excluded at 10 d.p.f. Scale bar represents 250 µm in A and B and 50 µm in C - E.

Dye exclusion experiments have long been used in rodent investigations into the integrity of the BBB [1,18–21]. The absence of dyes within the CNS following injection into peripheral vessels can be used as a marker of BBB integrity. We therefore used dyes of varying molecular weights to determine the developmental timecourse of size dependent exclusion from the zebrafish CNS. Dye exclusion experiments were initially performed on larvae from 2 to 30 d.p.f. and after initial analysis had narrowed down the relevant age range, studies were limited to larvae at 3, 4, 5, 8 and 10 d.p.f. Larvae were observed at 1 hour intervals after injection of the fluorescent dye into the pericardial cavity to determine when the fluorescent compound had diffused from the circulation into peripheral tissues (Figure 1A & B). Larvae were then anaethestised and fixed for cryosectioning. The fluorescent intensity of frozen sections of brain tissue was quantified for dye injected larvae and compared to saline injected siblings fixed at the same time point after injection. An example of the distribution and quantification process is shown in Figure 1C-E. In Evans blue (961 Da) injected zebrafish, fluorescence was observed in the brain at 3 d.p.f. but not at 5 d.p.f or subsequent stages (Figure 1F), suggesting that molecules of high molecular weight become excluded between 3 and 5 d.p.f. In contrast, exclusion of sodium fluorescein, a lower molecular weight compound (376 Da), was not observed until 10 d.p.f. (Figure 1G), suggesting a time dependent maturation relating to the size limit for molecules excluded from the CNS. 

### Expression of the zebrafish homologue of mammalian P-glycoprotein is developmentally regulated

**Figure 2:**
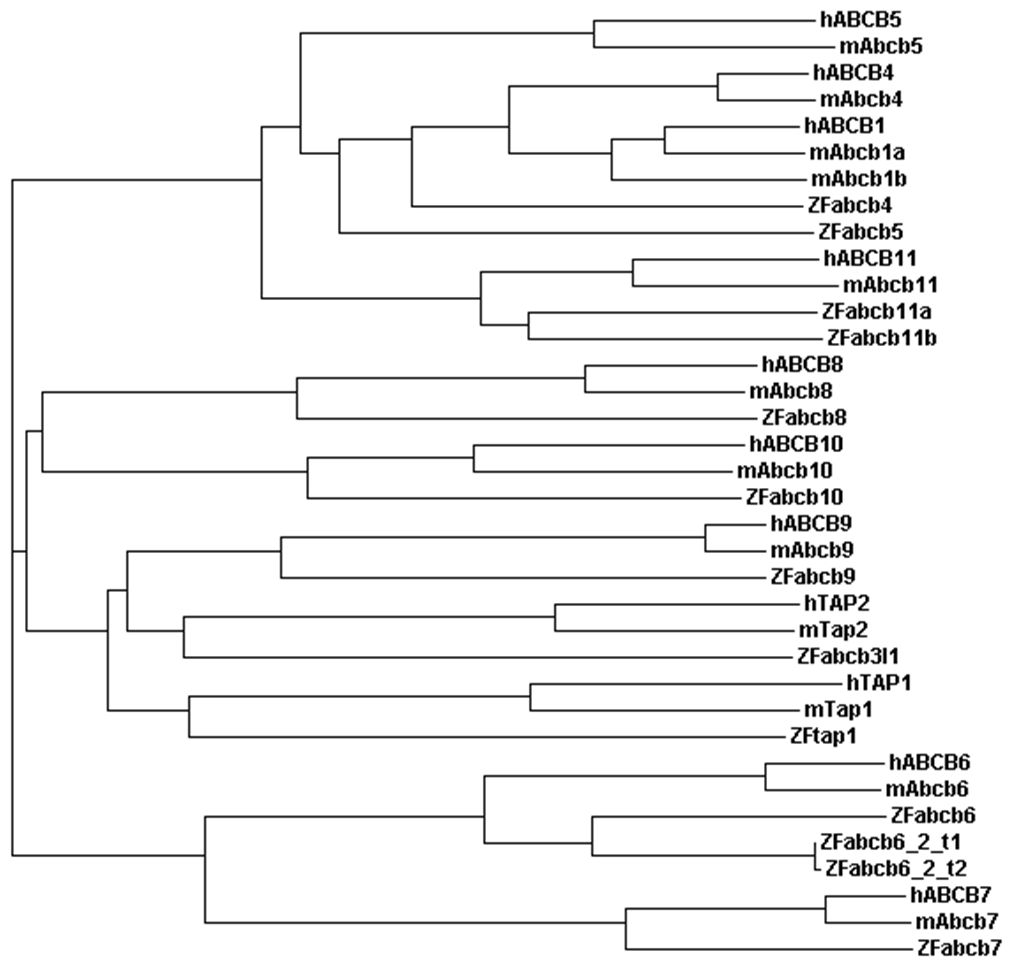
Phylogenetic analysis of mammalian and zebrafish *ABCB* genes. Phylogenetic analysis of the *ABCB* gene family in human, mouse and zebrafish. The zebrafish genome does not contain an annotated orthologue of mammalian *ABCB1*. Zebrafish *abcb4* and *abcb5* are identified as the closest homologues to mammalian *ABCB1, 4* and *5*.

Previous studies have reported that zebrafish, like rodents, have a duplicated *abcb1* (P-glycoprotein, Pgp, or multi-drug resistance) gene [22]. However, in the recent release of the zebrafish genome sequence (Genome assembly: Zv9), ENSDARG00000021787 is now annotated as *abcb5* (reported as *abcb1a* by [22]) and ENSDARG00000010936 as *abcb4* (reported as *abcb1b* by [22]). In man, the *ABCB* gene family comprises 11 members (*ABCB1, 4, 5, 6, 7, 8, 9, 10, 11, TAP1* and *TAP2*) whereas in zebrafish, *abcb1, abcb6* and *tap2* are missing, *abcb11* is duplicated (*abcb11a* and *abcb11b*) and, in addition, zebrafish possess abcb3l1. Phylogenetic analysis of the mammalian and zebrafish *abcb* family suggests that the zebrafish *abcb5* (ENSDARG00000021787) and *abcb4* genes (ENSDARG00000010936) are the closest homologues of mammalian ABCB1/4/5 (Figure 2) (see also Figure S1A and S1B for alignment of human, mouse and zebrafish Abcb4 and Abcb5 protein sequences). Peptide alignment analysis shows that zebrafish Abcb4 has 63% similarity at the amino acid level to human ABCB1 and 63% similarity to human ABCB4 (Table 1). The zebrafish abcb5 protein has 57% similarity at the amino acid level to human ABCB1 and 50% to human ABCB5 (Table 1). BLAST searches of the latest zebrafish genome release (Ensembl Zv9) with both human ABCB1 and mouse ABCB1A and ABCB1B identify zebrafish Abcb4 and Abcb5 as the closest zebrafish homologues to mammalian ABCB1. 

**Table 1:** Similarity between human, mouse and zebrafish ABCB1, ABCB4 and ABCB5 peptide sequences.

	Zebrafish Abcb4	Zebrafish Abcb5
Human ABCB1	63%	57%
Human ABCB4	63%	55%
Human ABCB5	54%	50%
Mouse ABCB1A	63%	56%
Mouse ABCB1B	61%	56%
Mouse ABCB4	63%	54%
Mouse ABCB5	52%	48%

Peptide sequences were aligned using ClustalW.

Since no specific orthologue of mammalian ABCB1 could be identified in the zebrafish genome (zv9), and since mammalian ABCB4 and ABCB5 are known to be expressed in the vascular endothelial cells of the CNS [1], we hypothesised that zebrafish Abcb4 or Abcb5 might function as efflux transporters in the vasculature surrounding the CNS and therefore investigated the expression of these proteins. 

**Figure 3:**
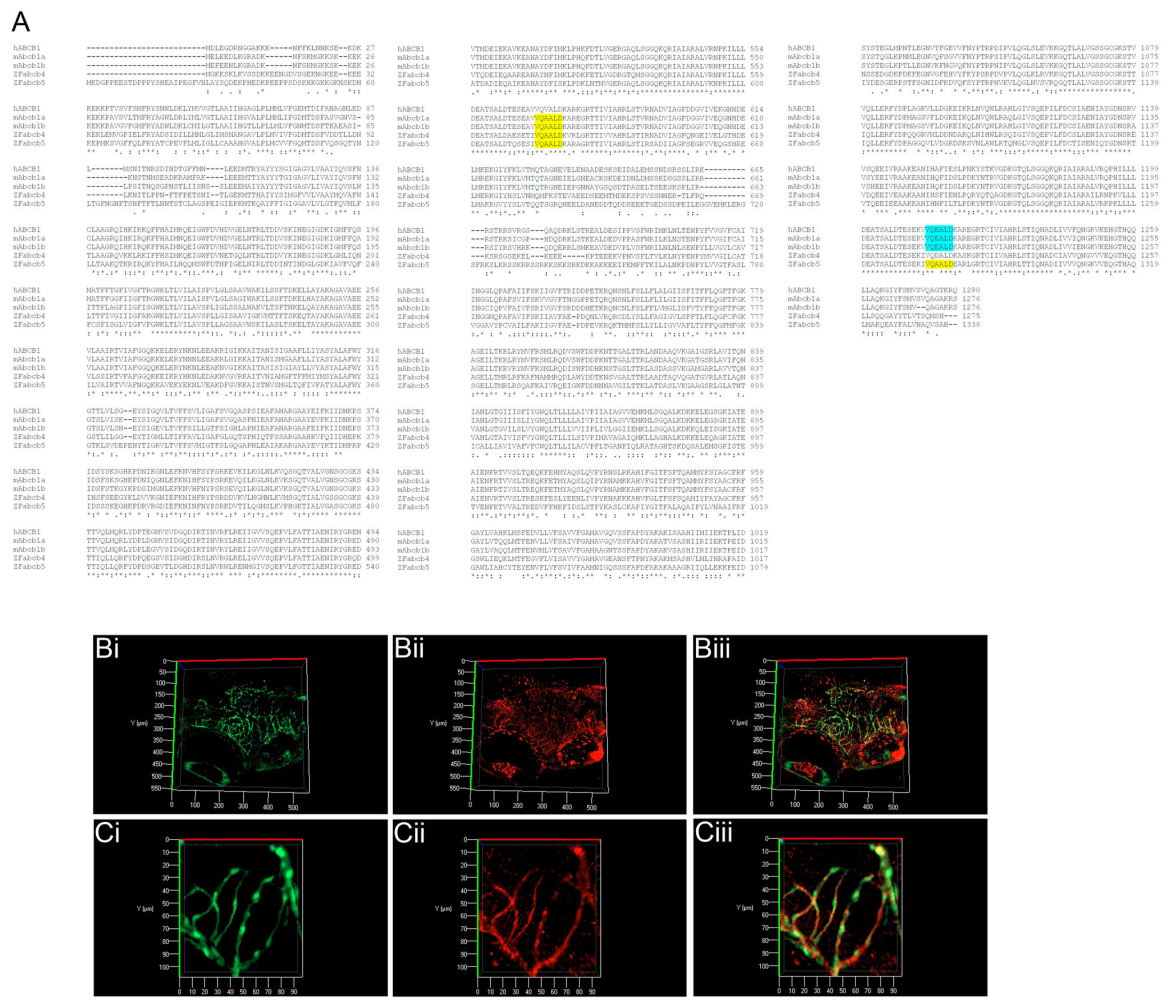
Sequence comparison and expression of zebrafish ABCB1/4/5 homologues. **A**) **Sequence alignment of human and mouse ABCB1 protein sequences with zebrafish Abcb4 and Abcb5**. **The zebrafish proteins show a high level of identity with the mouse and human ABCB1, ABCB4 and ABCB5 proteins**. **An antibody recognising the peptide sequences VQAALD (yellow) and VQEALD (blue) (Covance) was selected for use in zebrafish as the similarity of these peptides was conserved**. **B (low magnification) and C) (high magnification) 3D projections of optically sectioned wholemount Tg(fli1a:EGFP)^y1^ larvae stained with the anti-VQAALD antibody**. **Positive** **staining** **is** **observed** **in** **the** **vascular** **endothelium** **of** **the** **CNS** **at 8** **d.p.f , but** **not** **at** **earlier** **timepoints **(**see** Figure S2). **High magnification images (Ci-iii) demonstrate the co-localisation of ABCB1/4/5 antibody staining (red) on cerebral vessels (green)**. **Bi and Ci – GFP channel – maximum intensity projection of the cerebral vasculature of Tg(fli1a:EGFP)^y1^ transgenic larvae; Bii and Cii – Alexa 568 labelled antibody staining with ABCB1/4/5 antibody; Biii and Ciii – overlay**.

Using an antibody against a highly conserved amino acid sequence found in all mammalian Pgp isoforms [23] and also present in zebrafish Abcb4 and Abcb5 (Figure 3A), optical sections of wholemount Tg(fli1a:EGFP)^y1^ larvae at 10 d.p.f. revealed specific staining in the vascular endothelium of the CNS. A range of larval ages were then examined to determine the earliest time point with specific vascular staining. Staining was observed in the vascular endothelium of the CNS at 8 d.p.f. (Figure 3B & C) but not earlier (Figure S2). Based on phylogenetic analysis, peptide homology and protein expression, we assume that either the zebrafish *abcb5* (ENSDARG00000021787) or *abcb4* gene (ENSDARG00000010936) is the functional homologue of mammalian *ABCB1* and is hereafter referred to as *abcb1/4/5*.

### Expression of zebrafish Abcb1/4/5 correlates with the onset of active transport across the blood brain barrier

**Figure 4:**
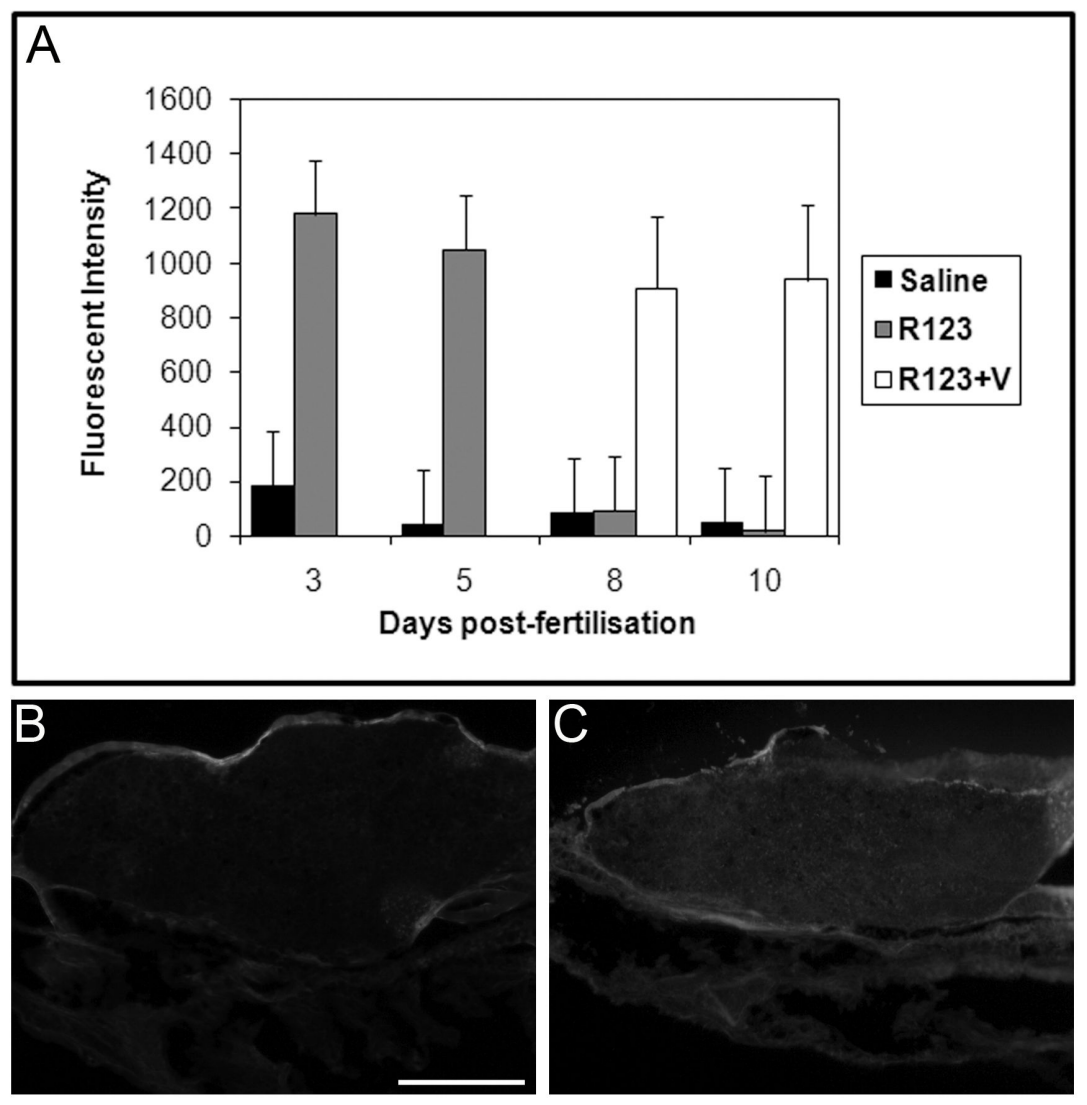
Rhodamine 123 distribution is altered in the presence of verapamil. A) Dye distribution and quantification experiments using rhodamine 123 (R123) were performed as described for Figure 1. Graph shows mean fluorescent intensity (± std dev.) for each treatment. Rhodamine 123 (grey bars), a substrate for mammalian ABCB1, ABCB4 and ABCB5, was excluded from the brain by 8 d.p.f. which, coincides with the onset of zebrafish Abcb/4/5 staining in the vasculature of the CNS. When 8 d.p.f. and 10 d.p.f. larvae were incubated with verapamil (white bars), an inhibitor of ABCB1, ABCB4 and ABCB5 in mammals, R123 failed to be excluded, consistent with blocked Abcb1/4/5 function. B and C) Representative images of parasagittal section of 5 d.p.f. larvae following pericardial injection of saline (B) or Rhodamine 123 (C). Fluorescence in the brain is observed following injection of Rhodamine 123. Scale bar represents 50 µm.

Dye exclusion experiments were performed with Rhodamine 123 (R123), a fluorescent substrate for ABCB1 [24], ABCB4 [25] and ABCB5 [26] in mammals, to investigate the function of Abcd1/4/5 during zebrafish development. R123 was excluded from the brain at 8 d.p.f., but not at 3 and 5 d.p.f (Figure 4). This time line coincides well with the expression of Abcb1/4/5 in the vascular endothelium of the CNS. To investigate whether the exclusion of R123 was indeed due to efflux mediated by a Abcb1/4/5 transporter, dye exclusion experiments were repeated in the presence of verapamil, a known inhibitor of mammalian ABCB1 [24], ABCB4 [25] and ABCB5 [27]. Larvae were incubated in 50 µg/ml verapamil prior to, during and after injection of R123 and analysed 3 hours after injection for distribution of R123 fluorescence. In the presence of verapamil, R123 penetrated into the brain also at 8 d.p.f. and 10 d.p.f. (Figure 4). Since R123 was excluded from the brain at these time points when incubated without verapamil, these results suggests that R123 is actively transported out of the brain by zebrafish Abcb1/4/5 from 8 d.p.f.

### The formation of a blood brain barrier correlates with developmental changes in drug distribution

A panel of 5 drugs known to either cross or not cross the BBB in mammals were selected to test in zebrafish larvae. Larvae of different ages were exposed to these compounds at non-toxic concentrations for 1 hour prior to collection of head and trunk tissue for analysis of drug concentration. A generic extraction method was developed and LC/MS/MS was performed to analyse the concentration of drug in head and trunk tissue in larvae of different ages. Drug/tissue concentrations were normalised against a standard and the relative amount of drug in head and trunk samples was compared to determine whether the drug distribution altered with larval age (Table 2). The amount of drug absorbed over the 1 hour exposure period varied greatly with some larval tissue containing as little as 5.9 % of the medium concentration for scopolamine N-butyl bromide and as much as 949% of the medium concentration for haloperidol, a variation that may reflect the hydrophobic/hydrophilic properties of the drugs tested. Indeed, there is a loose correlation between the partition coefficient (logP) value for each compound and the percentage of the drug absorbed from the medium. In addition, the absorption of each drug was found to vary between the different larval ages examined. This may reflect the maturation of different absorption routes, such as gut and gills and/or onset of expression of metabolising enzymes. 

**Table 2:** Age-dependent changes in distribution of drugs in zebrafish larvae.

**Drug (BBB penetration in mammals)**	**LogP^1^**	**Age (d.p.f.)**	**Absorption (whole body) (% uptake)**	**Head concentration (µg/g)**	**Trunk concentration (µg/g)**	**Trunk/Head ratio**
Scopolamine	0.98	5	-	-	-	-
(penetrant)		8	-	-	-	-
		10	12.8	.65	.63	1
Scopolamine N-butyl bromide	-1.11	5	7.2	.89	1.26	1.4
(excluded)		8	5.9	.88	.87	1
		10	11.3	1.47	1.93	1.3
Diphenhydramine	3.27	5	142.4	21.63	21.08	1
(penetrant)		8	184.1	31.21	24.03	0.8
		10	358.7	52.8	54.8	1
Haloperidol	4.3	5	732.6	115.5	104.29	0.9
(penetrant)		8	949.5	136.35	148.51	1.09
		10	713.5	112.7	101.35	0.9
Desloratadine (excluded)	3.2 (XlogP)	5	14.8	2.31	2.14	0.9
		8	39.1	2.57	3.29	1.3
		10	28.5	3.38	5.17	1.5
Desloratadine + verapamil		5	18.4	2.89	2.64	0.9
		8	7.9	1.17	1.2	1
		10	48.9	8.57	6.12	0.7

^1^ LogP values were obtained from ChemIDplus (http://chem.sis.nlm.nih.gov/chemidplus/); where LogP values were not available, XLogP values were obtained from PubChem (http://pubchem.ncbi.nlm.nih.gov/). Zebrafish larvae were exposed to 15 µg/ml of the test compound (5 µg/ml for scopolamine) for 1 hour and then collected for LC/MS/MS analysis. Absorption in whole larvae was calculated as percentage of drug concentration in the tissue compared to the medium. Drug concentrations from head and trunk tissue were calculated from duplicate samples and trunk/head ratio was used to highlight unequal tissue distribution as an indicator of BBB effects. Differences of greater than 20% (i.e. trunk/head ratio > 1.2) were considered to indicate lack of BBB penetration.

All drugs were equally distributed in head and trunk samples at 5 d.p.f., indicating the absence of a BBB at this age. Furthermore, diphenhydramine and haloperidol, which do cross the BBB in mammals, showed an equal distribution in head and trunk samples at all ages examined, indicating that these drugs also cross the zebrafish BBB. In scopolamine treated larvae, the drug level was below the detection limits at 5 and 8 d.p.f. However, scopolamine was detected at equal concentrations in head and trunk samples in 10 d.p.f. larvae, suggesting that scopolamine also crossed the BBB, as in mammals. In contrast, scopolamine N-butyl bromide and desloratadine, two drugs that do not cross the mammalian BBB, showed lower concentrations in the head versus the trunk at 10 d.p.f. and from 8 d.p.f. respectively, suggesting that these compounds are also excluded from the brain in zebrafish larvae. Exclusion of desloratadine at 8 d.p.f. correlates with the earliest expression of Abcb1/4/5 in the vascular endothelium. Since it is known that this drug is actively exported by Pgp (ABCB1) in mammals, we investigated whether the distribution of this drug would be altered by co-incubation with the ABCB1, ABCB4 and ABCB5 inhibitor, verapamil. Co-incubation with verapamil resulted in equalisation of the distribution of desloratadine in head and trunk samples at 8 d.p.f and an accumulation in the head versus the body at 10 d.p.f. Therefore, the exclusion of desloratadine from 8 d.p.f is dependent on the function of Abcb1/4/5 in zebrafish. 

### Anatomical features of a blood brain barrier

**Figure 5:**
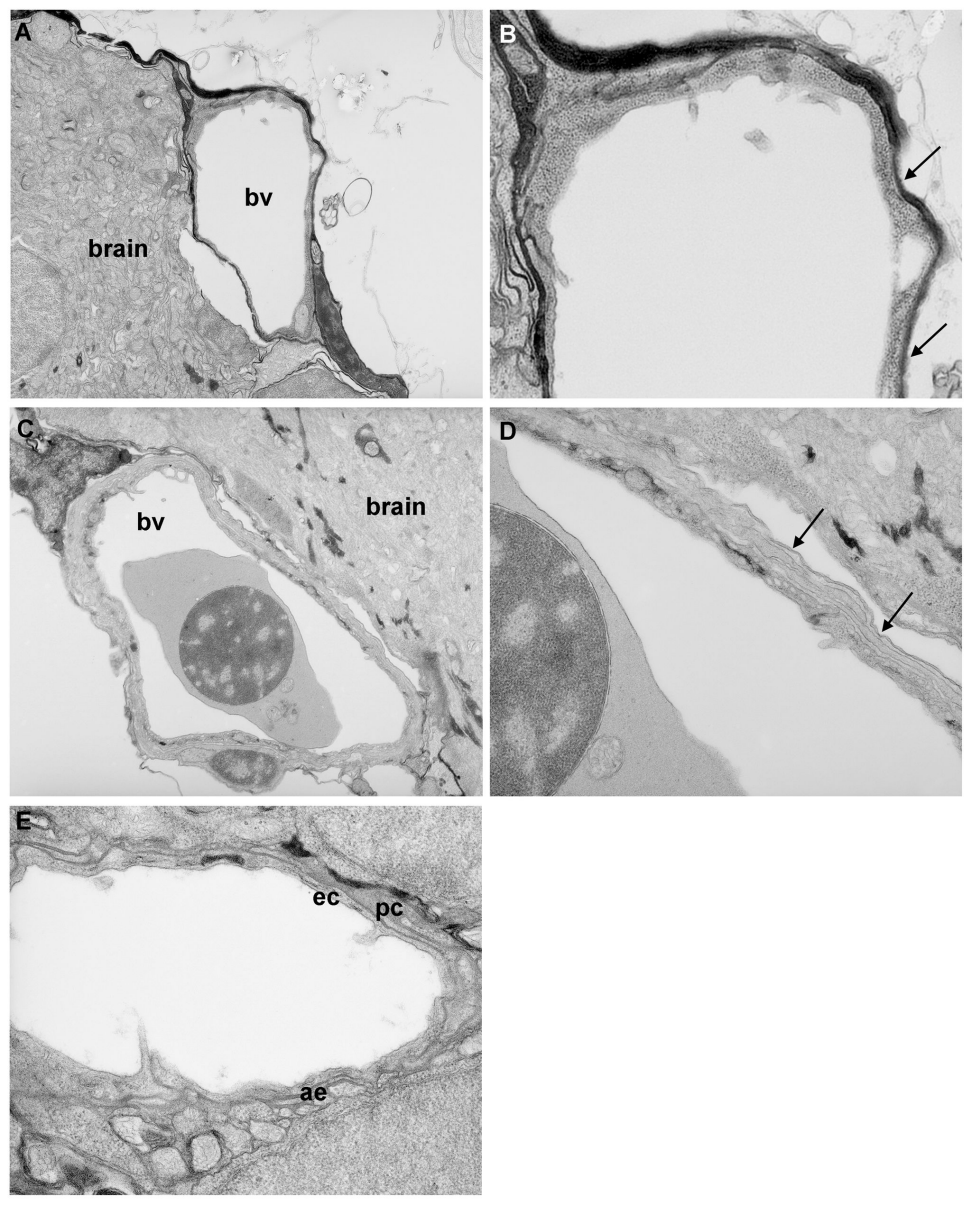
Transmission electron microscopy analysis of blood-brain barrier maturation. Parasaggital sections through the brain of zebrafish larvae at 3 d.p.f. and 10 d.p.f. A) At 3 d.p.f., blood vessels (bv) surrounding the brain are simple in structure. B) At high resolution, only a single membrane is observed (arrows) no evidence of double membranes was observed at any location examined. C) At 10 d.p.f., blood vessels surrounding the CNS are more complex in structure. D) At high resolution, a double layer membrane is apparent (arrows), indicative of the presence of tight junctions. E) In some vessels at 10 d.p.f., pericytes (pc) could be observed surrounding endothelial cells (ec). In addition, astrocyte endfeet (ae) were observed around some vessels at 10 d.p.f. but not at earlier ages. Magnification: A & C 5.2K; B, D & E 15.5K.

Having established that exclusion of small molecules from the zebrafish brain occurs between 3 and 10 d.p.f. (depending on the size of the molecule and the method of exclusion), we undertook an anatomical timecourse analysis using transmission electron microscopy (TEM) to determine whether there was also anatomical evidence for a BBB and if so, to characterise the maturation of these features. At 3 d.p.f., no evidence of tight junctions in the vessels around the brain and spinal cord was observed (Figure 5A & B). At 5 d.p.f., however, tight junctions were identified in some, but not all vascular endothelium (data not shown). By 10 d.p.f., double membranes were observed in the vascular endothelium at all sites investigated, suggesting that tight junctions are present by this age (Figure 5C & D). In addition, pericytes and astrocyte feet processes were observed in some sections at 10 d.p.f. (Figure 5E) and evidence for the latter was further supported by the co-localisation of glial fibrillary associated protein (GFAP) with GFP-positive vessels in the brains of Tg(fli1a:EGFP)^y1^ larvae at 10 d.p.f. but not earlier (Figure S3).

## Discussion

### The zebrafish BBB matures during larval development

Several studies have demonstrated that dyes or enzymatically active compounds are retained within cerebral microvessels of the larval zebrafish brain from as early as 3 d.p.f. [7,9–11], co-incident with the expression of the tight junction proteins ZO-1 and claudin5. However, these studies showed exclusion from only some, but not all cerebral vessels suggesting that there is not a fully functional BBB at this point. Here, we have combined anatomical analysis by TEM, functional investigation using fluorescent markers and compound uptake using LC/MS/MS to investigate the formation of the functional BBB in zebrafish and to demonstrate how this barrier matures during larval development. By administrating dyes of different molecular weights, we have shown that large molecular weight compounds are indeed excluded at 3 d.p.f. Although Evans blue is a fairly small molecule (961 Da), most of the dye becomes bound to serum albumin and has a size of >69kDa, therefore exclusion at 3 d.p.f. likely demonstrates exclusion of serum proteins and this is in keeping with the size exclusion findings from other studies [7,9–11]. By contrast, exclusion of sodium fluorescein (molecular weight of 376Da) does not take place until 10 d.p.f. indicating that the size dependent exclusion of molecules from the brain matures during larval development. 

### The zebrafish BBB possesses active transport systems

Active transport systems play a critical role in the function of the BBB in mammals. The most significant transporter in mammals is ABCB1 protein (Pgp), although other ABCB family transporters and organic anion transporters are also expressed by brain endothelial cells (reviewed in 1). These transporters have diverse but overlapping substrate specificities that help determine the partitioning of small molecules between the blood and the brain. A clinical example of the role of Pgp efflux is the non-sedating effects of second-generation antihistamines. Unlike the first generation counterparts (e.g. hydroxyzine, diphenhydramine and triprolidine), loratadine, desloratadine (the active metabolite of loratadine), and cetirizine (the active metabolite of hydroxyzine) are actively effluxed from the brain by Pgp and therefore do not have a sedative effect [28]. 

In mammals, ABCB1 is present in epithelial cells specialised in the secretion and excretion of undesired molecules, particularly in the gut, liver, kidney and BBB. Several different variants of ABCB5 are expressed in different tissues in humans; ABCB5.ts encodes the longest protein (1257 aa), believed to be a full transporter (i.e. the single protein functions as an efflux pump) with expression restricted to the testis; ABCB5 beta (812 aa) encodes a half-transporter which is only active as a dimer and expression is restricted to pigment cells. Evolutionary analysis of ABCB5 has shown that the ancestral gene is a full transporter and that ABCB5, 1, 4 and 11 share a common ancestor which began duplicating early in the evolution of the chordate lineage. Zebrafish have homologues of ABCB4 and ABCB5, but not ABCB1. The expression of zebrafish Abcb1/4/5 in the vasculature suggests that these proteins may fulfil the role of mammalian Pgp. By immunohistochemical and dye exclusion experiments, we have shown here both anatomical and functional evidence for active transport of molecules across the BBB in zebrafish. Abcb1/4/5 is first detected in the vascular endothelium of the CNS at 8 d.p.f. Thus whilst non-Pgp/ABCB5 substrate small molecules (e.g. sodium fluorescein) can penetrate the BBB and remain in the brain until day 10, Pgp/ABCB1/4/5 substrates (e.g. R123 and desloratidine) are actively effluxed from day 8.

### A zebrafish model for BBB compound penetration

The presence of the BBB has been one of the greatest obstacles in the development of drugs to treat neurological conditions, with less than 1% of small molecules penetrating this barrier. Various models have been developed to predict drug permeation through membranes. For example, measurements of drug partitioning in solvents is widely employed, although perfusion through solvents is not identical to diffusion across biological membranes and provides no information about active transport systems [29]. Indeed, as a result of transport proteins such as P-glycoproteins, the net distribution in the CNS of a variety of hydrophobic drugs, such as digoxin, cyclosporin and loperamide, is relatively low [30]. Models of BBB involving cell culture systems play some role in assessing whether drugs may cross the BBB, but are limited in that transport systems and blood-brain barrier enzymes may be severely down regulated or not present at all [31]. Furthermore, cells in such culture systems do not form rigid tight junctions.

Here, we have demonstrated that zebrafish larva may be used as an *in vivo* tool for the prediction of compound distribution. In accordance with other studies on compound uptake in larval zebrafish [32,33], we observed a loose correlation between the amount of compound absorbed and its logP value. The compound with the highest logP, haloperidol, showed highest accumulation in larvae and the compounds with low logP (scopolamine and n-butyl scopolamine) showed least absorption. However, 2 compounds with logP of approximately 3.2 (diphenhydramine and desloratidine) did not show a consistent pattern in the percentage of compound absorbed. This suggests that hydrophobic properties of a compound are not the sole determinant of their uptake into zebrafish and is in keeping with findings of previous studies [33]. Nevertheless, our study confirmed that all 5 compounds tested showed the same distribution in older zebrafish larvae as in mammals. An equal ratio of trunk/head drug distribution (i.e. trunk/head= 1.0) was observed at all ages for scopolamine, diphenhyramine and haloperidol, compounds known to cross the BBB in mammals. By contrast, ratios of greater than 1.2 were observed for scopolamine N-butyl bromide (at 10 d.p.f.) and desloratidine (at 8 and 10 d.p.f.), compounds that do not cross the mammalian BBB. In mammals, drug distribution is calculated as brain:plasma ratios. These studies show higher exclusion ratios than observed in zebrafish e.g. 1:8 for desloratadine and 1:20 for fexofenadine [34]. While this may suggest that the BBB may not yet be fully formed in larval zebrafish, our findings with dye exclusion assays would suggest otherwise. It is more likely that the lack of a more dramatic exclusion ratio is due to the exposure route (i.e. via immersion), or collection and analysis of whole head rather than brain tissue in the current study (in contrast to analysis on isolated brain and plasma samples in mammals). A more definitive analysis of compound distribution before and after BBB formation might be achieved by injection of compounds directly into the circulation. However, in our study, we sought to replicate the conditions used in standard zebrafish chemical screens, i.e. compound exposure by immersion, in order to determine whether the formation of the BBB limits the penetration of certain compounds into CNS tissue. These findings will therefore be of more relevance to groups performing compound screens with neurological and neurobehavioural endpoints and suggest that even following immersion (where absorption is thought to occur through the skin, gut and gills), compounds may be (partially) excluded from the CNS in older larvae. Since the 5 d.p.f. zebrafish larva is only 2-3 mm in length, brain dissection was not possible, and head samples therefore also contained non-CNS tissue such as skin, bone, muscle and gills. Nevertheless, our results suggest that changes from the 1:1 head:trunk concentration ratio can be used as an indicator of exclusion from the brain. These data would therefore suggest that zebrafish may be useful in determining whether a compound would penetrate the BBB *in vivo*, but not for accurate quantification. For example, analysis of compound uptake in head versus trunk at 5 d.p.f. and 10 d.p.f. could be used as a method for predicting BBB penetration. In addition, analysis of drug distribution at different larval ages (i.e. before and after the onset of Abcb1/4/5 expression) allows analysis of whether a particular drug is actively effluxed. Since the use of zebrafish screens to identify novel therapeutics is gaining popularity [35,36], these results allow for the rational screening of small molecules for CNS endpoints in zebrafish by appropriate age selection. 

In summary, the demonstration of a BBB in zebrafish larvae lends further weight to their importance as an experimental species for drug discovery, particularly for neurological indications. Furthermore, since the barrier is formed in larvae by 10 d.p.f., zebrafish may provide a rapid and accurate *in vivo* model for the prediction of BBB permeability for novel compounds.

## Supporting Information

Figure S1 **Alignment of human, mouse and zebrafish ABCB4 and ABCB5 protein sequences.** A) Alignment of human, mouse and zebrafish ABCB4 protein sequences. B) Alignment of human, mouse and zebrafish ABCB5 protein sequences. Peptide sequences were aligned using ClustalW.
(DOCX)

Figure S2 **Expression of zebrafish ABCB1/4/5 homologue at 3 d.p.f.** Maximum intensity projection of the cerebral vasculature of Tg(fli1a:EGFP)^y1^ transgenic embryos at 3 d.p.f. A) GFP labels the vasculature; B) Alexa 568 labelled antibody staining with ABCB1/4/5 antibody; C) overlay. ABCB1/4/5 antibody does not co-localise with the vascular endothelium at 3 d.p.f. although positive antibody staining is observed in the liver primordium (arrowhead).
(TIF)

Figure S3 **Expression of GFAP around cerebral vessels.** Maximum intensity projections of Tg(fli1a:EGFP)^y1^ transgenic larvae stained with GFAP antibody. A) At 7 d.p.f., GFAP staining is observed in the glia but does not co-localise with GFAP in the vasculature. B (low magnification) and C (high magnification) At 10 d.p.f., GFAP staining is observed in some cerebral vessels (arrowheads). Scale bar represents 50 µm.
(TIF)

Table S1 **LC conditions for haloperidol, desloratadine and diphenhydramine.**
(DOCX)

Table S2 **LC conditions for scopolamine and scopolamine N-butyl bromide.**
(DOCX)

Table S3 **MS Scan Parameters under APCI conditions.**
(DOCX)
