# Supplementary material for: Functional Characterisation of the Maturation of the Blood-Brain Barrier in Larval Zebrafish
Source: PLoS One. 2013 Oct 16;8(10):e77548. doi: 10.1371/journal.pone.0077548 (PMC3797749; doi:10.1371/journal.pone.0077548)
Supplement: Figure S1 — Alignment of human, mouse and zebrafish ABCB4 and ABCB5 protein sequences. A) Alignment of human, mouse and zebrafish ABCB4 protein sequences. B) Alignment of human, mouse and zebrafish ABCB5 protein sequences. Peptide sequences were aligned using ClustalW. (DOCX) [file pone.0077548.s001.docx]

**Figure S1**

**A)**

hABCB4 MDLEAAKNGTAWRPTSAEGDFELGISSKQKRKKTKTVKMIGVLTLFRYSDWQDKLFMSLG 60

mAbcb4 MDLEAARNGTARR---LDGDFELGSISNQGREKKKKVNLIGLLTLFRYSDWQDKLFMFLG 57

ZFabcb4 MGKKSKLKVSSDK-KEENGDVSGEKNGKEEKEEKEKLEMVGPIELFRYADSIDILLMMLG 59

*. :: : :: : :**.. .:: :::.:.::::* : ****:* * *:* **

hABCB4 TIMAIAHGSGLPLMMIVFGEMTDKFVDT-----AGNFSFPVNFSLSLLNPGKILEEEMTR 115

mAbcb4 TLMAIAHGSGLPLMMIVFGEMTDKFVDN-----TGNFSLPVNFSLSMLNPGRILEEEMTR 112

ZFabcb4 LIMSMANGAVLPLMVIVFGDMTDSFVDDTLLDNLKNITLPPNFTFPETS-NITLGEKMTT 118

:*::*:*: ****:****:***.*** *:::* **::. . . * *:**

hABCB4 YAYYYSGLGAGVLVAAYIQVSFWTLAAGRQIRKIRQKFFHAILRQEIGWFDINDTTELNT 175

mAbcb4 YAYYYSGLGGGVLVAAYIQVSFWTLAAGRQIKKIRQKFFHAILRQEMGWFDIKGTTELNT 172

ZFabcb4 HAIYYSIMGFVVLVAAYMQVAFWTLAAGRQVKKLRKIFFHSIMKQEIGWFDVNETGQLNT 178

:* *** :* ******:**:*********::*:*: ***:*::**:****:: * :***

hABCB4 RLTDDISKISEGIGDKVGMFFQAVATFFAGFIVGFIRGWKLTLVIMAISPILGLSAAVWA 235

mAbcb4 RLTDDVSKISEGIGDKVGMFFQAIATFFAGFIVGFIRGWKLTLVIMAISPILGLSTAVWA 232

ZFabcb4 RLTDDVYKINEGIGDKLGMLIQNLTTFIVGIIIGFAKGWKLTLVILAVSPLLGISAAVIG 238

*****: **.******:**::* ::**:.*:*:** :********:*:**:**:*:** .

hABCB4 KILSAFSDKELAAYAKAGAVAEEALGAIRTVIAFGGQNKELERYQKHLENAKEIGIKKAI 295

mAbcb4 KILSTFSDKELAAYAKAGAVAEEALGAIRTVIAFGGQNKELERYQKHLENAKKIGIKKAI 292

ZFabcb4 KVMTTFTSKEQTAYAKAGAVAEEVLSSIRTVFAFGGQKKEIKRYHKNLEDAKNVGVRKAI 298

*::::*:.** :***********.*.:****:*****:**::**:*:**:**::*::***

hABCB4 SANISMGIAFLLIYASYALAFWYGSTLVISKEYTIGNAMTVFFSILIGAFSVGQAAPCID 355

mAbcb4 SANISMGIAFLLIYASYALAFWYGSTLVISKEYTIGNAMTVFFSILIGAFSVGQAAPCID 352

ZFabcb4 TVNIAMGFTFFMIYMSYALAFWYGSTLILGGEYTIGMLLTIFFAVLIGAFGLGQTSPNIQ 358

:.**:**::*::** ************::. ***** :*:**::*****.:**::* *:

hABCB4 AFANARGAAYVIFDIIDNNPKIDSFSERGHKPDSIKGNLEFNDVHFSYPSRANVKILKGL 415

mAbcb4 AFANARGAAYVIFDIIDNNPKIDSFSERGHKPDNIKGNLEFSDVHFSYPSRANIKILKGL 412

ZFabcb4 TFSSARGAAHKVFQIIDHEPKINSFSEEGYKLDVVKGNIEFKNIHFRYPSRDDVKVLNGM 418

:*:.*****: :*:***::***:****.*:* * :***:**.::** **** ::*:*:*:

hABCB4 NLKVQSGQTVALVGSSGCGKSTTVQLIQRLYDPDEGTINIDGQDIRNFNVNYLREIIGVV 475

mAbcb4 NLKVKSGQTVALVGNSGCGKSTTVQLLQRLYDPTEGKISIDGQDIRNFNVRCLREIIGVV 472

ZFabcb4 NLKVMSGQTIALVGSSGCGKSTTIQLLQRFYDPQEGSVSIDGHDIRSLNVRGLRELIGVV 478

**** ****:****.********:**:**:*** **.:.***:***.:**. ***:****

hABCB4 SQEPVLFSTTIAENICYGRGNVTMDEIKKAVKEANAYEFIMKLPQKFDTLVGERGAQLSG 535

mAbcb4 SQEPVLFSTTIAENIRYGRGNVTMDEIEKAVKEANAYDFIMKLPQKFDTLVGDRGAQLSG 532

ZFabcb4 SQEPVLFATTIAENIRYGRQDVTQDEIEQAAREANAYNFIMKLPDKFETLVGDRGTQMSG 538

*******:******* *** :** ***::*.:*****:******:**:****:**:*:**

hABCB4 GQKQRIAIARALVRNPKILLLDEATSALDTESEAEVQAALDKAREGRTTIVIAHRLSTVR 595

mAbcb4 GQKQRIAIARALVRNPKILLLDEATSALDTESEAEVQAALDKAREGRTTIVIAHRLSTIR 592

ZFabcb4 GQKQRIAIARALVRNPKILLLDEATSALDAESETIVQAALDKVRLGRTTIVVAHRLSTIR 598

*****************************:***: *******.* ******:******:*

hABCB4 NADVIAGFEDGVIVEQGSHSELMKKEGVYFKLVNMQTSGS-QIQSEEFELN--DEKAATR 652

mAbcb4 NADVIAGFEDGVIVEQGSHSELMKKEGIYFRLVNMQTAGS-QILSEEFEVELSDEKAAGD 651

ZFabcb4 NADVIAGFQNGEIVELGTHDELMERKGIYHSLVNMQMFKSTEVAEEDSEEMTMDEKSPSV 658

********::* *** *:*.***:::*:*. ***** * :: .*: * ***:.

hABCB4 MAPNGWKSRLFRHSTQKNLKNSQMCQKSLDVETDGLEANVPPVSFLKVLKLNKTEWPYFV 712

mAbcb4 VAPNGWKARIFRNSTKKSLKSP--HQNRLDEETNELDANVPPVSFLKVLKLNKTEWPYFV 709

ZFabcb4 SSMN--ERTLFRQKSRSGSEKE-----LKEEEKPTEEEKVPNVSFLTVLKLNYPEWPYMV 711

: * : :**:.::.. :. : *. : :** ****.***** .****:*

hABCB4 VGTVCAIANGGLQPAFSVIFSEIIAIFGPGDDAVKQQKCNIFSLIFLFLGIISFFTFFLQ 772

mAbcb4 VGTVCAIANGALQPAFSIILSEMIAIFGPGDDAVKQQKCNMFSLVFLGLGVLSFFTFFLQ 769

ZFabcb4 VGILCATINGGMQPAFAVIFSKIIAVFAEPDQNLVRQRCDLYSLLFAGIGVLSFFTLFLQ 771

** :** **.:****::*:*::**:*. *: : :*:*:::**:* :*::****:***

hABCB4 GFTFGKAGEILTRRLRSMAFKAMLRQDMSWFDDHKNSTGALSTRLATDAAQVQGATGTRL 832

mAbcb4 GFTFGKAGEILTTRLRSMAFKAMLRQDMSWFDDHKNSTGALSTRLATDAAQVQGATGTRL 829

ZFabcb4 GFCFGKAGELLTMRLRFKAFNAMMRQDLAWYDDTKNSVGALTTRLAADTAQVQGATGVRL 831

** ******:** *** **:**:***::*:** ***.***:****:*:********.**

hABCB4 ALIAQNIANLGTGIIISFIYGWQLTLLLLAVVPIIAVSGIVEMKLLAGNAKRDKKELEAA 892

mAbcb4 ALIAQNTANLGTGIIISFIYGWQLTLLLLSVVPFIAVAGIVEMKMLAGNAKRDKKEMEAA 889

ZFabcb4 ATLAQNVANLGTAIVISFVYGWQLTLLILSIVPIMAVAGAIQMKLLAGHALKDKKELEQA 891

* :*** *****.*:***:********:*::**::**:* ::**:***:* :****:* *

hABCB4 GKIATEAIENIRTVVSLTQERKFESMYVEKLYGPYRNSVQKAHIYGITFSISQAFMYFSY 952

mAbcb4 GKIATEAIENIRTVVSLTQERKFESMYVEKLHGPYRNSVRKAHIYGITFSISQAFMYFSY 949

ZFabcb4 GKIATEAIENVRTVVSLTRESKFESLYEENLIVPYKNAKKKAHVFGLTFSFSQAMIYFAY 951

**********:*******:* ****:* *:* **:*: :***::*:***:***::**:*

hABCB4 AGCFRFGAYLIVNGHMRFRDVILVFSAIVFGAVALGHASSFAPDYAKAKLSAAHLFMLFE 1012

mAbcb4 AGCFRFGSYLIVNGHMRFKDVILVFSAIVLGAVALGHASSFAPDYAKAKLSAAYLFSLFE 1009

ZFabcb4 AGCFKFGSWLIEQKLMTFEGVFLVISAVVYGAMAVGEANSFTPNYAKAKMSASHVLMLIN 1011

****:**::** : * *..*:**:**:* **:*:*.*.**:*:*****:**:::: *::

hABCB4 RQPLIDSYSEEGLKPDKFEGNITFNEVVFNYPTRANVPVLQGLSLEVKKGQTLALVGSSG 1072

mAbcb4 RQPLIDSYSGEGLWPDKFEGSVTFNEVVFNYPTRANVPVLQGLSLEVKKGQTLALVGSSG 1069

ZFabcb4 RAPAIDNSSEDGDKPDKFEGNVGFEHVYFKYPSRPDVPVLQGLKLRVKKGQTLALVGSSG 1071

* * **. * :* ******.: *:.* *:**:*.:*******.*.**************

hABCB4 CGKSTVVQLLERFYDPLAGTVFVDFGFQLLDGQEAKKLNVQWLRAQLGIVSQEPILFDCS 1132

mAbcb4 CGKSTVVQLLERFYDPMAGSV-------LLDGQEAKKLNVQWLRAQLGIVSQEPILFDCS 1122

ZFabcb4 CGKSTTIQLLERFYDPQQGRV-------MLDDNDAKQLNIHWLRSQIGIVSQEPVLFDCS 1124

*****.:********* * * :**.::**:**::***:*:*******:*****

hABCB4 IAENIAYGDNSRVVSQDEIVSAAKAANIHPFIETLPHKYETRVGDKGTQLSGGQKQRIAI 1192

mAbcb4 IAENIAYGDNSRVVPHDEIVRAAKEANIHPFIETLPQKYNTRVGDKGTQLSGGQKQRIAI 1182

ZFabcb4 LAENIAYGDNSREVDQEEIVEAAKAANIHSFIENLPQRYQTQAGDKGTQLSGGQKQRIAI 1184

:*********** * ::*** *** ****.***.**::*:*:.*****************

hABCB4 ARALIRQPQILLLDEATSALDTESEKVVQEALDKAREGRTCIVIAHRLSTIQNADLIVVF 1252

mAbcb4 ARALIRQPRVLLLDEATSALDTESEKVVQEALDKAREGRTCIVIAHRLSTIQNADLIVVI 1242

ZFabcb4 ARAILRNPKVLLLDEATSALDTESEKIVQDALDKASKGRTCIIVAHRLSTIQNADCIAVV 1244

***::*:*::****************:**:***** :*****::*********** *.*.

hABCB4 QNGRVKEHGTHQQLLAQKGIYFSMVSVQAGTQNL 1286

mAbcb4 ENGKVKEHGTHQQLLAQKGIYFSMVNIQAGTQNL 1276

ZFabcb4 QNGVVVEQGTHQQLLSQQGAYYTLVTSQMSH--- 1275

:** * *:*******:*:* *:::*. * .

**B)**

hABCB5 ---------------------------------MENSERAEEMQENYQRNGT--AEEQ-P 24

mAbcb5 ---------------------------------MANSERTNGLQETNQRYGP--LQEQVP 25

ZFabcb5 MKDGPPEESTDPPPYSHEAIPEGFVNLAYSQDEKPHEDKPEEPPSDSKHKGKKGKNSKDN 60

:.::.: . :: * :.:

hABCB5 KLRKEAVGSIEIFRFADGLDITLMILGILASLVNGACLPLMPLVLGEMSDNLIS------ 78

mAbcb5 KVGNQAVGPIEIFRFADNLDIVLMTLGILASMINGATVPLMSLVLGEISDHLIN------ 79

ZFabcb5 KEPMKSVGFFQLFRYATCPEVFLMLIGLLCAAAHGVALPLMCVVFGQMTDSFVQSGQTYN 120

* ::** :::**:* :: ** :*:*.: :*. :*** :*:*:::* ::.

hABCB5 -----GCLVQTNTTNYQNCTQSQEK-----LNEDMTLLTLYYVGIGVAALIFGYIQISLW 128

mAbcb5 -----GCLVQTNRTKYQNCSQTQEK-----LNEDIIVLTLYYIGIGAAALIFGYVQISFW 129

ZFabcb5 LTGFNGNFTSNFTFTLNNTSTCLAGSPEIGIEPKMTKQAYFFIGIGGAVLVLGTFQVMLF 180

* :... . :* : :: .: : :::*** *.*::* .*: ::

hABCB5 IITAARQTKRIRKQFFHSVLAQDIGWFDSCDIGELNTRMTDDIDKISDGIGDKIALLFQN 188

mAbcb5 VITAARQTTRIRKQFFHSILAQDISWFDGSDICELNTRMTGDINKLCDGIGDKIPLMFQN 189

ZFabcb5 LLTAAKQTKRIRQKYFHAILHQQMSWFDTHPIGELNIRLTDDINTINDGLGDKIAVFVQF 240

::***:**.***:::**::* *::.*** * *** *:*.**:.: **:****.::.*

hABCB5 MSTFSIGLAVGLVKGWKLTLVTLSTSPLIMASAAACSRMVISLTSKELSAYSKAGAVAEE 248

mAbcb5 ISGFSIGLVISLIKSWKLSLVVLSTSPLIMASSALCSRMIISLTSKELDAYSKAGAVAEE 249

ZFabcb5 FCSFISGLVIGFVFGWKLTLVILAVSPLLAGSAAVWSKILASLTSKELTAYAKAGAVAEE 300

:. * **.:.:: .***:** *:.***: .*:* *::: ******* **:********

hABCB5 VLSSIRTVIAFRAQEKELQRYTQNLKDAKDFGIKRTIASKVSLGAVYFFMNGTYGLAFWY 308

mAbcb5 ALSSIQTVTAFGAQEKEIQRYTQHLKDAKDAGIKRATASKLSLGAVYFFMNGAYGLAFWY 309

ZFabcb5 ILVAIRTVVAFNGQKKAVEKYEKNLVEAKDFGVKKAISTNVSMGLTQFIVFATYALAFWY 360

* :*:** ** .*:* :::* ::* :*** *:*:: ::::*:* . *:: .:*.*****

hABCB5 GTSLILNGEPGYTIGTVLAVFFSVIHSSYCIGAAVPHFETFAIARGAAFHIFQVIDKKPS 368

mAbcb5 GTSLIFGGEPGYTIGTILAVFFSVIHSSYCIGSVAPHLETFTVARGAAFNIFQVIDKKPN 369

ZFabcb5 GTKLSVDEPENYTIGRVLTVFFSVMIGTFSLGQGAPNLEAIAKARGAAYEVYKTIDMPRP 420

**.* .. .**** :*:*****: .::.:* .*::*::: *****:.:::.**

hABCB5 IDNFSTAGYKPESIEGTVEFKNVSFNYPSRPSIKILKGLNLRIKSGETVALVGLNGSGKS 428

mAbcb5 IDNFSTAGFVPECIEGNIEFKNVSFSYPSRPSAKVLKGLNLKIKAGETVALVGPSGSGKS 429

ZFabcb5 IDSSSKEGHKPDRVRGDIEFKNINFNYPSRKDVTILQGMSLKVPHGKTIALVGASGCGKS 480

**. *. *. *: :.* :****:.*.**** . .:*:*:.*:: *:*:**** .*.***

hABCB5 TVVQLLQRLYDPDDGFIMVDENDIRALNVRHYRDHIGVVSQEPVLFGTTISNNIKYGRDD 488

mAbcb5 TTVQLLQRLYDPEDGCITVDENDIRAQNVRHYREQIGVVRQEPVLFGTTIGNNIKFGREG 489

ZFabcb5 TTIQLLQRFYDPDSGEVTLDGHDIRSLNVRWLRENMGIVSQEPVLFGTTIAENIRYGRED 540

*.:*****:***:.* : :* :***: *** *:::*:* **********.:**::**:.

hABCB5 VTDEEMERAAREANAYDFIMEFPNKFNTLVGEKGAQMSGGQKQRIAIARALVRNPKILIL 548

mAbcb5 VGEKEMEQAAREANAYDFIMAFPKKFNTLVGEKGAQMSGGQKQRIAIARALVRNPKILIL 549

ZFabcb5 ATDADIEQAIKEANAYDFISKLPDKLNTMVGERGAQLSGGQKQRIAIARALVKNPKILLL 600

. : ::*:* :******** :*.*:**:***:***:***************:*****:*

hABCB5 DEATSALDSESKSAVQAALEKASKGRTTIVVAHRLSTIRSADLIVTLKDGMLAEKGAHAE 608

mAbcb5 DEATSALDTESESLVQTALEKASKGRTTIVVAHRLSTIRGADLIVTMKDGMVVEKGTHAE 609

ZFabcb5 DEATSALDTQSESIVQAALDKARAGRTTIVIAHRLSTIRSADIIAGFSEGRVVEQGSHRE 660

********::*:* **:**:** ******:********.**:*. :.:* :.*:*:* *

hABCB5 LMAKRGLYYSLVMSQDIKKADEQMESMTYSTERKTN------------------------ 644

mAbcb5 LMAKQGLYYSLAMAQDIKKVDEQMESRTCSTAGNAS------------------------ 645

ZFabcb5 LMAKKGVYYSLVTQQTSGRQNEELDANEDDTQDDSEEETGEDSSDPEILEGGVEMKLERG 720

****:*:****. * : :*:::: .* .:.

hABCB5 ----SLPLHSVKSIKSDFIDKAEESTQSKEISLPEVSLLKILKLNKPEWPFVVLGTLASV 700

mAbcb5 ----YGSLCDVNSAKAPCTDQLEEAVHHQKTSLPEVSLLKIFKLSKSEWPFVVLGTLASA 701

ZFabcb5 SFRKSLKRSSKRRSSRKKSKKSRKDKKAKKEEIPEMPFTKILALNKPDWPYLVVGTFASL 780

. . . .: .: : :: .:**:.: **: *.*.:**::*:**:**

hABCB5 LNGTVHPVFSIIFAKIITMFGNNDKTTLKHDAEIYSMIFVILGVICFVSYFMQGLFYGRA 760

mAbcb5 LNGSVHPVFSIIFGKLVTMFEDKNKATLKQDAELYSMMLVVLGIVALVTYLMQGLFYGRA 761

ZFabcb5 VGGAVYPCVAILFAKIIGVFAEPDPEVKRQKTMMFSLLYLLIGVVAFLTYFFQGFMFGKS 840

:.*:*:* .:*:*.*:: :* : : . ::.: ::*:: :::*::.:::*::**:::*::

hABCB5 GEILTMRLRHLAFKAMLYQDIAWFDEKENSTGGLTTILAIDIAQIQGATGSRIGVLTQNA 820

mAbcb5 EENLAMRLRHSAFKAMLYQDMAWYDDKENNTGALTTTLAVDVAQIQGAATSRLGIVTQDV 821

ZFabcb5 GELLTMRLRSQAFKAIVRQEIGWFDDNNNAVGILTTKLATDASLVKGAAGSRLGLATNTI 900

* *:**** ****:: *::.*:*:::* .* *** ** * : ::**: **:*: *:

hABCB5 TNMGLSVIISFIYGWEMTFLILSIAPVLAVTGMIETAAMTGFANKDKQELKHAGKIATEA 880

mAbcb5 SNMSLSILISFIYGWEMTLLILSFAPVLAVTGMIQTAAMAGFANRDKQALKRAGKIATEA 881

ZFabcb5 CALLIAVIVAFVFCWQLTLLILACVPFLTGANFIQLRATAGHTSKDQSALEMSGKISTET 960

: ::::::*:: *::*:***: .*.*: :.:*: * :*.:.:*:. *: :***:**:

hABCB5 LENIRTIVSLTREKAFEQMYEEMLQTQHRNTSKKAQIIGSCYAFSHAFIYFAYAAGFRFG 940

mAbcb5 VENIRTVVSLTRERAFEQMYEETLQTQHRNALKRAHITGCCYAVSHAFVHFAHAAGFRFG 941

ZFabcb5 VENFKTVVALTREDVFFHKFIDSLSTPYKASLCKAPIYGITFALAQAIPYLVNAAIFRFG 1020

:**::*:*:**** .* : : : *.* :: : :* * * :*.::*: ::. ** ****

hABCB5 AYLIQAGRMTPEGMFIVFTAIAYGAMAIGETLVLAPEYSKAKSGAAHLFALLEKKPNIDS 1000

mAbcb5 AYLIQAGRMMPEGMFIVFTAIAYGAMAIGETLVWAPEYSKAKAGASHLFALLKNKPTINS 1001

ZFabcb5 AWLIAHCYTEYENVFLVFSVIVFAAMNIGQSSSFAPDFAKAKAAAGRIIQLLEKKPEIDI 1080

*:** *.:*:**:.*.:.** **:: **:::***:.*.::: **::** *:

hABCB5 RSQEGKKPDTCEGNLEFREVSFFYPCRPDVFILRGLSLSIERGKTVAFVGSSGCGKSTSV 1060

mAbcb5 CSQSGEKPDTCEGNLEFREVSFVYPCRPEVPVLQNMSLSIEKGKTVAFVGSSGCGKSTCV 1061

ZFabcb5 YDESGERPSTFSGNIDFKDVQFSYPTRPNVKVLQGLNVSVRQGQTLALVGSSGCGKSTTI 1140

.:.*::*.* .**::*::*.* ** **:* :*:.:.:*:.:*:*:*:********** :

hABCB5 QLLQRLYDPVQGQVLFDGVDAKELNVQWLRSQIAIVPQEPVLFNCSIAENIAYGDNSRVV 1120

mAbcb5 QLLQRFYDPMKGQVLLDGVDVKELNVQWLRSQTAIVSQEPVLFNCSIAENIAYGDNSRMV 1121

ZFabcb5 QLLERFYDPAGGQVLVDGRDSKSVNLAWLRTQMGLVSQEPILFDCTISENIQYGDNSRTV 1200

***:*:*** ****.** * *.:*: ***:* .:*.***:**:*:*:*** ****** *

hABCB5 PLDEIKEAANAANIHSFIEGLPEKYNTQVGLKGAQLSGGQKQRLAIARALLQKPKILLLD 1180

mAbcb5 PLEEIKEVADAANIHSFIEGLPRKYNTLVGLRGVQLSGGQKQRLAIARALLRKPKILLLD 1181

ZFabcb5 TQEEIEEAAKKANIHNFILTLPDKYNTRVGDKGTQLSGGQKQRIAIARALVRKPKLLLLD 1260

. :**:*.*. ****.** ** **** ** :*.*********:******::***:****

hABCB5 EATSALDNDSEKVVQHALDKARTGRTCLVVTHRLSAIQNADLIVVLHNGKIKEQGTHQEL 1240

mAbcb5 EATSALDNESEKVVQQALDKARRGKTCLVVAHRLSTIQNADMIVVLQNGSIKEQGTHQEL 1241

ZFabcb5 EATSALDTESEKIVQAALDEARLGRTCIVIAHRLTTIQNADIIVVVQNGKVVEQGTHAQL 1320

*******.:***:** ***:** *:**:*::***::*****:***::**.: ***** :*

hABCB5 LRNRDIYFKLVNAQSVQ- 1257

mAbcb5 LRNGDTYFKLVAAH---- 1255

ZFabcb5 MAKQEAYFALVNAQVSAH 1338

: : : ** ** *:
