# Supplementary figures and images for: Functional Characterisation of the Maturation of the Blood-Brain Barrier in Larval Zebrafish
Source: PLoS One. 2013 Oct 16;8(10):e77548. doi: 10.1371/journal.pone.0077548 (PMC3797749; doi:10.1371/journal.pone.0077548)

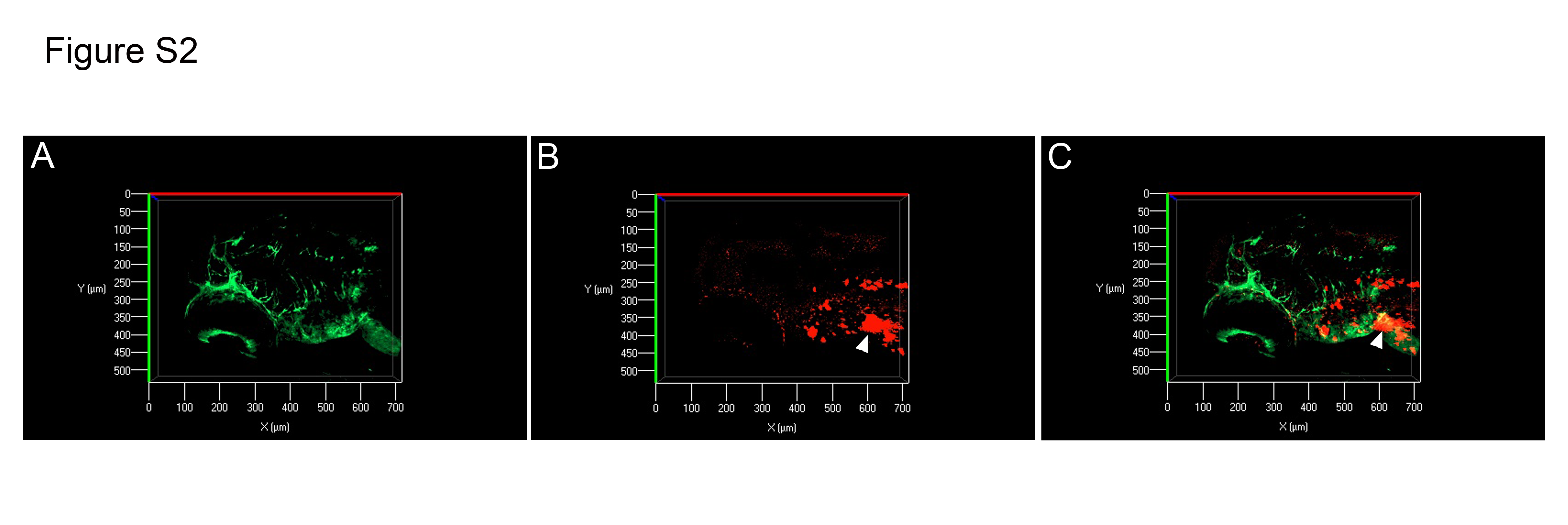

Supplement: Figure S2 — Expression of zebrafish ABCB1/4/5 homologue at 3 d.p.f. Maximum intensity projection of the cerebral vasculature of Tg(fli1a:EGFP)y1 transgenic embryos at 3 d.p.f. A) GFP labels the vasculature; B) Alexa 568 labelled antibody staining with ABCB1/4/5 antibody; C) overlay. ABCB1/4/5 antibody does not co-localise with the vascular endothelium at 3 d.p.f. although positive antibody staining is observed in the liver primordium (arrowhead). (TIF) [file pone.0077548.s002.tif]

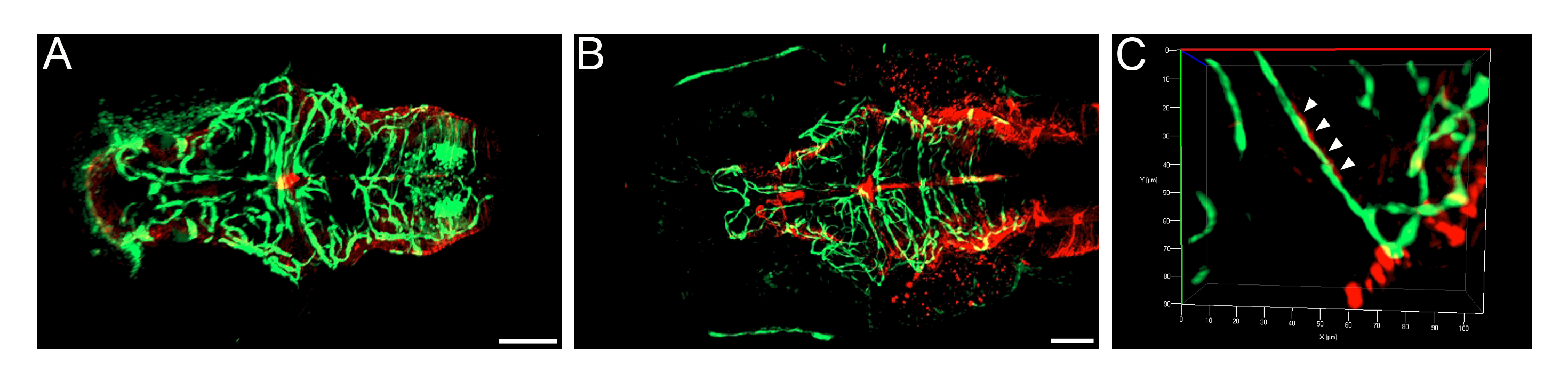

Supplement: Figure S3 — Expression of GFAP around cerebral vessels. Maximum intensity projections of Tg(fli1a:EGFP)y1 transgenic larvae stained with GFAP antibody. A) At 7 d.p.f., GFAP staining is observed in the glia but does not co-localise with GFAP in the vasculature. B (low magnification) and C (high magnification) At 10 d.p.f., GFAP staining is observed in some cerebral vessels (arrowheads). Scale bar represents 50 µm. (TIF) [file pone.0077548.s003.tif]
