# Supplementary material for: Functional Characterisation of the Maturation of the Blood-Brain Barrier in Larval Zebrafish
Source: PLoS One. 2013 Oct 16;8(10):e77548. doi: 10.1371/journal.pone.0077548 (PMC3797749; doi:10.1371/journal.pone.0077548)
Supplement: Table S1 — LC conditions for haloperidol, desloratadine and diphenhydramine. (DOCX) [file pone.0077548.s004.docx]

**Table S1. LC conditions for haloperidol, desloratadine and diphenhydramine.**

| **Time (minutes)** | **% acetic acid (0.1%)** | **% acetonitrile** | **Flow Rate mLmin^-1^** |
| --- | --- | --- | --- |
| 0.0 | 95 | 5 | 1.0 |
| 3.5 | 50 | 50 | 1.0 |
| 5.0 | 30 | 70 | 1.0 |
| 6.0 | 0 | 100 | 1.0 |
| 6.5 | 95 | 5 | 1.0 |
