# Supplementary material for: Functional Characterisation of the Maturation of the Blood-Brain Barrier in Larval Zebrafish
Source: PLoS One. 2013 Oct 16;8(10):e77548. doi: 10.1371/journal.pone.0077548 (PMC3797749; doi:10.1371/journal.pone.0077548)
Supplement: Table S3 — MS Scan Parameters under APCI conditions. (DOCX) [file pone.0077548.s006.docx]

**Table S3. MS Scan Parameters under APCI conditions**

| **Drug** | **[M+H]^+^** | **CE** | **Isolation Width** |
| --- | --- | --- | --- |
| Haloperidol | 377.0 | 50 | 4.0 |
| Scopolamine | 304.1 | 36 | 2.0 |
| Diphenhydramine | 255.9 | 28 | 2.0 |
| Desloratadine | 311.1 | 35 | 4.1 |
